# Admitting the heterogeneity of social inequalities: intersectionality as a (self-)critical framework and tool within mental health care

**DOI:** 10.1186/s13010-023-00144-6

**Published:** 2023-11-24

**Authors:** Florian Funer

**Affiliations:** Institute of Ethics and History of Medicine, Eberhard Karls University Tübingen, Tübingen, Germany

**Keywords:** Intersectionality, Heterogeneity, Stigma, Vulnerabilities, Discrimination, Social inequality, Mental health, Psychiatry, Mental health care

## Abstract

Inequities shape the everyday experiences and life chances of individuals at the margins of societies and are often associated with lower health and particular challenges in accessing quality treatment and support. This fact is even more dramatic for those individuals who live at the nexus of different marginalized groups and thus may face multiple discrimination, stigma, and oppression. To address these multiple social and structural disadvantages, intersectional approaches have recently gained a foothold, especially in the public health field. This study makes an empirically informed argument for the merits of increasing the use of intersectional frameworks in the mental health field. In the mental health field, the potential for greater attention to multiple unjustified disadvantages appears to be of particular importance, as many mental health service users already face stigma and discrimination because of their mental health issues and thus may benefit particularly frequently and far-reachingly from effective problem awareness about multiple disadvantages. Intersectional approaches may help address the complexity, interdependence, and mutual constitution of social inequalities better than previous approaches that examined only one category of sociostructural stratification. By helping to identify the needs of those at the greatest risk of poor health, intersectional frameworks and tools can contribute not only to better address the needs of multiple disadvantaged individuals with mental health issues but also to the promotion of equity in the field of mental health, contributing to the reduction of health disparities.

## Introduction

Intersectionality has become a crucial concept in understanding the complexities of social identity and inequality in various fields, including health care. Coined by Kimberlé W. Crenshaw in 1989 [1], intersectionality refers to the different ways in which multiple personal characteristics, such as *race*,^1^ ethnicity, gender, sexual orientation, social class, age, and abilities, interact to shape individuals’ experiences in society and everyday life. In contrast to approaches that examine socially salient characteristics and their effects as isolated entities, intersectional approaches foreground the many different layers of social inequalities that can combine in unique ways to disadvantage people.

Recently, the analytic potential of intersectional approaches has also been discovered in the health field, particularly in public health [4–6]. This is mainly because health inequities are commonly associated with membership in socially marginalized groups and empirical findings regarding those particularly affected by health inequities could be particularly helpful to inform policy-making [7]. The relevance of social determinants to the maintenance of health and the development of diseases as well as the effects of social stratification and stigmatization on health along categories, such as *race*, ethnicity, gender, and socioeconomic status, are well documented [8, 9]. However, to date, in most cases, only the effects of single social categories have been studied for health [10], for instance, the influence of *race*-related stressors on mental health [11].

In the mental health field, intersectionality could shed light on the ways in which mental health disparities and inequalities are not solely based on individual-level factors, such as biology or behaviour, but are also influenced by broader social and structural forms of discrimination, stigma and oppression, such as systemic racism, sexism, homophobia, classism, ageism, and (dis-)ableism. A deeper understanding of the complex interaction of discrimination, stigma and oppression due to membership in multiple socially marginalized groups and how they affect mental health seems particularly promising, as individuals with “mental illness” are already more likely to face unjustified disadvantages due to their disorder [8, 10, 12–17]. Nevertheless, most of the available research does not consider the effects and implications of membership or attribution to multiple stigmatized social groups among people with mental health issues [18]. Future engagement to understand intersectional disadvantages may lead to more appropriate intervention and support services in the mental health field and thus contribute to the reduction of mental health disparities.

In this paper, I will argue for the potential of intersectional approaches for mental health research, policy, and practice and illustrate the ways in which mental health professionals can adopt an intersectional lens to surface social and structural barriers and forms of discrimination, stigma and oppression, thereby better meeting the health care needs of people with diverse conditions and contributing to promote health equity. To demonstrate this, the first step is to briefly outline the relevance of social inequalities and their effects on mental health. In the second step, mental health issues are introduced as distinct social category that makes unjustified inequalities more likely. Attention will be given primarily to the stigmatizing effects associated with membership or assignment of individuals to the social group of people with mental health issues. In the third step, intersectionality is presented as a suitable framework that does better justice to the complexity of disparities and social injustice than previous approaches. In the fourth step, the value of intersectionality as a framework and tool particularly within mental health care practice is outlined, which calls for health care professionals to be critically aware of the possibility of multiple social disadvantages in dealing with people with mental health issues, particularly recognizing the responsibility for mental health issues and their successful treatment less than before in the individual and more in social and structural causes. Taking this shift in responsibility seriously might have far-reaching implications for mental health care practices. Finally, a brief summary concludes the paper.

## Social inequalities and their effects on mental health

Before going into more detail about the incremental value of an intersectional framework, the relevance of social inequalities for mental health care should first be elucidated. Here, not all disparities in mental health care are of importance: Fundamentally, there can be differences between persons who require different – and thus proper – treatment. The disparities of interest from an ethical point of view are not those that are justified by clinical appropriateness, needs, or preferences but rather those that result from social or structural inequalities [16, 19]. While for the former, restrictions on rights (e.g., to access specific treatments and resources) may be morally justified due to the lack of need and/or preference for treatment, the latter constitute baseless discrimination, regardless of any need and/or preference [20]. Inequality, that is, unjustified discrimination against individuals across social categories, has a significant impact on mental health, as people from marginalized communities are not only more likely to suffer from mental health problems and to receive psychiatric diagnoses but also often face multiple barriers to accessing necessary mental health services and resources or simply receive poorer quality of treatment.

In the discourse, those social categories have become established as axes of analysis for which there is reliable evidence that belonging to or having identity characteristics of such a category may lead significantly more likely to unjustified disparities in certain social contexts. There are at least two forms of “membership” to a social group that may be statistically associated with negative, but sometimes different, disadvantages: On the one hand, an individual may experience unjustified adverse disparities as a member of a particular group *because of that membership* (e.g., belonging to the group of people with low socioeconomic status due to low socioeconomic status). On the other hand, an individual may face unjustified adverse disparities because of the *presence of certain publicly perceived or assumed characteristics* by which membership in a particular group is inferred (e.g., belonging to the low socioeconomic status group based on one’s physical appearance or behaviour). While the former represents a membership corresponding to the case, the latter is merely a spurious attribution of membership – it should be kept in mind, however, that both forms can lead to disadvantages and are not always unambiguous, especially in the context of mental health issues.

Mental health disparities have largely been documented for categories of social stratification, such as *race*, ethnicity, gender, sexual orientation, and socioeconomic status [8, 9].^2^ For example, for some time, it is known that members of racial or ethnic minorities may face additional barriers to accessing the mental health care system [11, 21–23]. This may be due to higher levels of *race*-associated stigma, insufficiently culturally skilled mental health care professionals, and/or distrust in the health care system. Prejudice, negative beliefs, and a lack of information about mental disorders and their causes within a cultural community can also discourage people from accessing mental health care and continuing treatment [23, 24]. These disparities can be further exacerbated by discriminatory policies, practices, and attitudes within social, legal, and health systems, which often perpetuate already existing social inequalities. For instance, depending on the social system and public access to free or low-cost treatment, the socioeconomic status of a person plays a decisive role in regard to the possibilities of receiving (psycho-)therapy appropriate to the disorder, which may be time- and resource-consuming [21]. As one consequence, persons of minority populations tend to have a higher burden of their disease. It has been shown that although members of minority populations have a lower likelihood of experiencing acute episodes of major depressive disorder (MDD) compared to “Caucasians”^3^ [25], they have a higher likelihood of experiencing prolonged, chronic, and severely debilitating depression, which can heavily impact their daily life [25]. In addition, less access to and poorer quality of treatment are quite plausible explanations for the increased persistence of disorders such as posttraumatic stress disorder (PTSD) or alcoholism among, for example, indigenous populations [21]. Negative effects on mental health have also been attributed to other social and structural forms of discrimination and oppression within rules, policies and institutional procedures that arbitrarily restrict the rights of some people as well as the sequels of historical trauma, such as colonialism, racism, and sexism [26, 27].

## Mental health issues as a distinct category of social stratification due to stigmatization

In addition to the more familiar social categories such as *race*, ethnicity, gender, sexual orientation, and socioeconomic status, there are calls for additional consideration of other categories such as educational attainment, employment, marital/parental status, (dis-)abilities or age – and probably many of which I am not aware.^4^ The inclusion of “mental health issues” or “mental illness” as distinct category requires justification of the extent to which membership in this group, or the presence of identity characteristics by which membership in this group is ascribed, may cause or at least facilitate unjustified discrimination.^5^ Here, it should be borne in mind that the terms “mental illness” or “mental health issues” refer to a wide range of different conditions and difficulties that individuals may face with regard to their mental well-being. Thus, the use of this broad category sometimes obscures the differences between mental health issues that also exist, as not all of these issues are associated with the same degree and forms of discrimination.

Crucial to efforts aimed at demonstrating the longer-term disadvantages of persons with mental health issues were the initial efforts of Morton Birnbaum [28] and the successive academic elaborations of Michael L. Perlin (cf. “sanism” [29]) and Judi Chamberlin (cf. “mentalism” [30, 31]). Considering the research efforts of the last few decades, demonstrating a strong correlation between (ascribed) membership in the group of people with mental health issues and unjustified discrimination has become an easy task. Discrimination against individuals with mental health issues may be understood as the result of stigma, that is, stereotypes, prejudices, and discrimination against individuals with mental health issues (see as fundamental works [32] and [33] and for the stigma of mental health issues [16, 18, 34–39]). People with mental health issues “are devalued, rejected, shamed, and excluded based on a socially discredited health condition.” ([16], p. 4) The potency of mental health issue stigma may vary in multiple social contexts, such as among individuals with certain cultural backgrounds [23]. Mostly, although not exclusively, they are based on misconceptions or misinformation that guide perceptions, attitudes, and behaviour towards members of the social group [18], which is primarily the case for the attitudes and behaviours of other people [40]. However, subsequently, it often becomes also adopted in the form of an individual’s self-stigma, that is, the anticipation of negative behaviour towards oneself [41, 42].^6^

One of the flawed assumptions mentioned in the literature concerns the degree of an individual’s own responsibility for the development and maintenance of mental health issues, such as substance dependence disorders [43] or eating disorders [44]. Here, the illnesses are conceived as self-induced or as the result of personal – and thus largely avoidable – decisions in the past. Interestingly, it has been shown that even in such cases where biomedical causes as well as causative childhood trauma as not self-inflicted causes were assumed, nonetheless, the only presence of mental health issues seemed to be associated with higher stigma [45]. A possible explanation offered for the case of substance dependence disorders by White (2001) (cf. [46] cited by [43]) is that, even in cases where the individual is seen as a victim of his or her disorder, it is assumed by others that the disorder would be associated with certain behaviours (e.g., lack of impulse control, unreliability, dangerousness, etc.) and that the disorder in general weakens their values and good intentions.

Another problem is the generalization of very specific (negative) assumptions, which may be valid for single individuals of the group, to the collective of persons labelled as “mentally ill”. However, given the multifaceted nature of disorders, manifestations, severities, and treatment successes in different individuals with mental health issues, a general loss of competence or deviance of behaviour may not be assumed based solely on the label “mentally ill” [20]. Corrigan et al. (2004) therefore pointed out the double hurdle of people with mental health issues: “Mental illness strikes with a two-edged sword. On one hand, people must struggle with the symptoms and disabilities that prevent them from achieving many of their life goals. On the other, the stigma of mental illness further hampers their opportunities and aspirations.” ([20], p. 489).

Mental illness stigma can influence different health outcomes in significant ways [47]. Experiencing stigma because of mental health issues is a barrier to seeking or using mental health treatment and social support, resulting in delayed help-seeking and more frequent treatment discontinuations or withdrawals [10, 18, 23, 34, 35, 37]. For example, eating disorder stigma correlates with negative effects on individuals’ psychological well-being as well as their treatment-seeking behaviour [44]. The stigma of mental illness also seems to (co-)cause harmful effects, for instance, due to prolonged processes of recovery and social reintegration [48] or to make the development of physical comorbidities that remain untreated (e.g., diabetes, heart disease, stroke, and epilepsy) [23, 49], unemployment and suicidality more likely [41].

Accumulating evidence over the past several decades illustrates that stigma due to mental health issues is experienced in almost all domains of life, not the least of which is health care itself [21, 50, 51]. In fact, a particularly strong entanglement of mental health issue stigma and the system of health care institutions can be shown, insofar as the latter contribute to the reproduction of stigma [16, 51–53]. Additionally, individuals with mental health issues, even when they access treatments, are more likely to experience negative attitudes toward them from health care professionals, adverse health care interactions, and overuse of coercive and paternalistic situations [16, 51].

This different behaviour in seeking and accessing treatment and support as well as the different quality of treatment translates concretely into health disparities, including excess morbidity and early mortality among individuals with mental health issues [54]. Found by research, people living with mental health issues show a life expectancy that is 10 to 25 years shorter than that of other people [55, 56].

The stigmatization and self-stigmatization of individuals suffering from mental health issues are known and have been denounced already for a long time; their importance has also been recognized and taken up by, for example, the World Health Organization [57] or the American National Academies for Sciences, Engineering and Medicine [58]. However, they still impact the treatment and identity construction as well as the social and structural conditions. The means of choice to counteract stigmas, in addition to facilitating access to treatment, strengthening more integral care coordination, and monitoring structural stigma [16], are mostly efforts to eliminate or at least reduce misconceptions and misinformation through education with the aim to improve attitudes and practices towards people with mental health issues [16, 37, 45, 51]. Crucially, however, as with other socially acquired attitudes and thought patterns regarding *race*, ethnicity, gender, sexual orientation, or class, they often implicitly – and therefore persistently – shape the beliefs of individuals, even when a person’s knowledge may change as a result of an educational campaign. Perhaps for this reason, it has been shown in adults that contact-to-contact experiences appear to have a greater impact on reducing stigma [18, 36]. Addressing this challenge, therefore, requires sustained instruments of critical reflection that explicitly make stigmas of different categories their objects.

## Intersectionality – one step further to analyse the complexity of social inequalities

The literature on the effects of social stratification and their implications for mental health, of which only a very small part is highlighted here, is extensive. Most investigations on health disparities in recent decades have focused on one category of social stratification [13]. However, Pachankis et al. (2017), for example, showed within their sample that subjects with mental health issues had an average of six stigmatized conditions [59]. Regardless of the exact number of axes for which inequities can be demonstrated, it will have to be acknowledged that for any of these disparities it is the case that they lack moral justification by clinical appropriateness, existing needs, or treatment preferences of those affected. Because people may sometimes face multiple barriers and unjustified inequalities, “framing disparities along single axes of social inequality can obscure the excess risk faced by populations at the nexus of multiple marginalized social categories” [13].

The fact that advantages and disadvantages are not caused by only one but sometimes by multiple social identity characteristics of an individual has mostly been interpreted within cumulative frameworks [60–62]. Gary (2005), for example, described with the term “double stigma” the experience of mental illness in members of four ethnic minority groups in the United States [23]. It was assumed that members of ethnic minority groups are already confronted with prejudice and discrimination because of their membership in this group but that these prejudices and discrimination can be further exacerbated by additional mental health issues [23]. In such “double-disadvantage theories” [18], the disadvantages of several social determinants are accumulated so that the joint effect on mental health is understood to be equivalent to the sum of their respective effects (e.g., ethnicity + gender + mental disorder; cf. [8]). Approaches such as this have led to an increased awareness of the double burden of stigmatization of people with mental health issues and, for example, HIV or obesity [45].

Nevertheless, such approaches fail to recognize the potentially existing salience of certain identity characteristics in comparison to others and the potential emergence of further effects. Here, salience refers to the “prominence” [18] of one characteristic to which certain behaviours are more likely to be attributed than to other characteristics, the significance of which in turn recedes into the background in the perception of the counterpart or the public. This mechanism is understood less as an accusation of social ignorance than as an admission to an efficient but simplistic way of dealing with complexity in everyday social life. Depending on the prevailing perception in a society, for example, a person’s *race*, ethnicity, gender, socioeconomic status or mental disorder may be particularly prominent and thus predispose to stigmatization more than other characteristics. Other marginalized social identity characteristics may therefore be given little attention, even though this would actually be necessary to avoid discrimination on their basis. In some cases, different social identity characteristics even seem to partially mediate each other [60].

With Oexle and Corrigan (2018), we can assume that these effects (double-disadvantage and prominence) are not mutually exclusive, but rather that they “fluctuate depending on the number and types of intersecting social group memberships, their visibility, and contextual factors (including cultural setting and perceiver characteristics)” [18]. However, considering only one of the two effects does not take seriously the unique interlocking relationships that can exist across social identity characteristics [5, 8, 63]. We cannot be reduced to just one identity or one constructed characteristic, but are members of or have identity characteristics of several social groups.

It is precisely this limitation that is addressed conceptually by intersectional approaches. They shift attention to the simultaneously present and mutually constitutive effects of different social identity characteristics on individuals’ experiences and life chances [8]. Initially, applied as a theoretical framework for qualitative studies in law, sociology, and psychology [13], intersectional approaches seek to elucidate how individuals experience multiple forms of systemic oppression, such as racism, classism, sexism, and homophobia, as well as their interactions [6, 13].^7^ Intersectional approaches recognize the multifaceted reality of identities and their social contexts within which some disadvantages may accumulate or reinforce each other, some may become prominent, some may recede into the background, and others may also mutually mediate each other. The experience of a particular social identity characteristic, such as being LGBTQIA + , may differ across the presence of other social identity characteristics, such as socioeconomic status, *race*, ethnicity or mental illness (for the latter cf. for example [64]). Differentiating intersections that exist, whereby constraints and privileges may operate concurrently [5, 8], recognizes the finely chiselled reality of discrimination (experiences) in different social contexts better than previous approaches have been able to – without claiming to have reached the end of the road.

That the implications of intersectional approaches for the mental health field have not been widely acknowledged until recently was demonstrated, for example, by the in matter important publication of the National Academies of Sciences, Engineering and Medicine entitled “*Ending Discrimination Against People with Mental and Substance Use Disorders: The Evidence for Stigma Change*” (2016) [58], which still addresses “double and intersecting stigmas” [ibid.] within a short paragraph by highlighting their mere existence. However, knowing the great influence of social determinants on the development and progress of mental disorders as well as on the chances of their successful treatment (see Sect. 2), an intersectional approach can support a more nuanced understanding of the diversity of mental health service users and their needs as “people[s]-in-context” [65]. This contextuality of individuals also means analysing the relevance of known social categories in the respective context and not prematurely assuming that certain identity characteristics are fundamentally disadvantageous. Just as the significance of one’s own sexual orientation can differ significantly depending on the nation in which one lives, other characteristics such as mental health issues may also have a different significance depending on the respective living environment. The understanding of the impact of intersecting social categories on mental health, diagnosis, and treatment is still limited. However, the first results regarding mental health point to “paradoxical patterns of stratification” [8], which underline the importance of intersectional approaches, as the different probabilities for certain mental health conditions and progressions in different subgroups cannot simply be derived a priori from already established theoretical approaches.

For example, using an intersectional framework that explicitly considers gender, *race*, class, and ethnicity, between-group variations in attention deficit hyperactivity disorder (ADHD) diagnosis can be determined [8]. The authors found that male, high-income, white, non-Hispanic children from low-education backgrounds were more likely to receive a diagnosis – which is at least one prerequisite for sufficient treatment – than their Hispanic counterparts [ibid.]. In another intersectionality-framed study [12], it was shown that compared to white heterosexual women, white sexual minority women had higher risks for depressive symptoms, alcohol use, tobacco use, and marijuana use; while Black sexual minority women had higher tobacco and marijuana use only [12].

As I have tried to illustrate thus far, intersectional approaches may be useful for better describing the complexity of disparities, effects of stigma and discrimination in different social contexts. If this is the case, there are good reasons from an ethical point of view as to why intersectional approaches should play a greater role in mental health practice in the future than they have done thus far.

## Seeing mental health service users in context: The value of integrating individual’s social identities and existing structures of oppression in mental health research and treatment

Mental health disparities across different social categories are avoidable, even if the identification, precise description, and elimination of their causes may be difficult [13]. If the goal of health equity for all members of social communities is pursued, it entails a commitment to reduce and ultimately eliminate unjustified inequalities and their social determinants. With Braveman (2014), this goal can be summarized as follows: “Pursuing health equity means striving for the highest possible standard of health for all people and giving special attention to the needs of those at greatest risk of poor health, based on social conditions.” [66] An intersectional lens might contribute to the pursuit of this goal in general but in the mental health field in particular if it finds consideration in research, policy, and practice.

A closer look at intersections reveals the complexity of stigmas and their negative consequences on mental health service users’ experiences and opportunities, not least on their capabilities to achieve or pursue certain health outcomes. This reveals the unique needs of individuals in different social categories, which may differ significantly from those in the statistical middle of a group. Insights from research on social inequalities clearly reject the primacy of reductionist models of biomedical or lifestyle or behavioural causation of illness based on the assumption of individual agency over matters of health [8, 67]. The emphasis of recent decades on the highest possible specificity in identifying disease mechanisms and their treatment seems unable to meet the goal of health equity, given the concurrent rapid increase in social and structural inequalities and their massive impact on mental health. Such an objective does progressively less justice to disorders such as schizophrenia, anxiety, and depression, so that health equity seems to become increasingly remote [68]. Intersectional approaches, therefore, focus compensatory on the structural and social preconditions and obstacles to the maintenance of (mental) health as well as an individual’s chances to take up support and treatment options despite all social barriers. Intersectionality should not be seen as an alternative to the above approaches but as an addendum because it brings to the surface the conditions, difficulties and barriers, knowing that they fundamentally impact a person’s agency as well as her help-seeking, self-esteem, and social functioning, which in turn can have a lasting impact on her mental health and well-being. Uncovering these social and structural barriers and forms of oppression helps to better address the health needs of people with different conditions and contexts. Taking such a conceptual framework of intersectionality seriously, which localizes the responsibility for one’s own mental health issues as well as for the use of support and therapy services less in the individual’s pathophysiological predisposition or behaviour and more in the social and structural determinants and life circumstances, would probably have far-reaching consequences for research and treatment approaches to address stigmatization, discrimination and oppression, among which there has been thus far a predominance of behavioural interventions rather than social and structural interventions [10].

The role of social and structural environmental conditions is by no means a novelty in mental health practice but is in fact deeply inscribed in it. For example, as one consequence of the biopsychosocial understanding of illness introduced at its time, the multiaxial system of the *Diagnostic and Statistical Manual of Mental Disorders* III (DSM-III) of 1980 and the subsequent version DSM-IV of 1994 explicitly required the contextual status of a person (Axis IV) to make a diagnosis. Moreover, the intensive efforts of activist movements of people with mental health issues led to a critical reflection on the stigmatizing effects and power structures inherent in the mental health care system and, as a result, to international reforms. By no means should the importance of social and structural factors be considered less for other health fields and medical disciplines, but it may be assumed that the mental health field is likely to be a pioneer in considering factors beyond the individual patient and his or her constitution. However, intersectional insights into the complex synergies and interdependencies of stigma effects highlight the markedly heterogeneous needs of individuals, of which, for example, male, high-income, white, Hispanic children from low-education backgrounds seem to require different enabling measures for adequate ADHD diagnosis than their non-Hispanic counterparts [8]. Such a finer granularity of collectives belonging to different social identities, once it has been well documented, allows for the development of quality therapy and support options tailored to the real needs of this patient group and thus a more appropriate addressing of health disparities [5, 13].

In addition to the critical function that intersectional approaches in the mental health field may assume vis-à-vis existing social injustices at the sociostructural level, they could also assume a forward-looking self-critical function at the individual level during the process of psychiatric diagnoses. To date, the heterogeneity of social and structural conditions, interpretation patterns and probably associated effects, in the context of which a diagnosis of mental illness is made, plays only a minor role, if any, in the process of making that diagnosis. However, these contextual conditions either promote or impede the opportunities that may arise from a diagnosis of mental illness for the person affected [69]. While members of certain groups may be particularly empowered by the diagnosis and the treatment and support opportunities which are opened by it, others – presumably especially members of already marginalized groups – may need more wide-ranging or quite different support measures to benefit equally from the same diagnosis rather than merely experiencing additional disadvantages. As Bergey et al. (2022) recently highlighted with the example of ADHD diagnosis, research with an intersectional framework might help to explore how a diagnosis is interpreted in different contexts and with which effects these interpretations are associated: “[F]or some, a diagnosis might be viewed as a mechanism of social control, while for others it could be seen as a gateway for much-needed intervention and potential improvements in quality of life.” [8] The diagnosis is not an end in itself. Anticipating the social and structural opportunities and potential benefits and harms of a diagnosis for a person who is already marginalized by other social categories may contribute to a more responsive diagnosis and increased outcomes for that person. The making of a diagnosis must therefore be assessed in advance with regard to the stigma potentially accepted by diagnosing the individual’s mental health issues.

Intersectional frameworks and tools, if further developed in the future, may thus make a critical contribution that could benefit mental health practice in at least two ways: On the one hand, they raise awareness of the importance of sociostructural preconditions of health and its maintenance as well as existing disparities, undermining one-sided biomedical or behaviour-associated explanations of disease causation, of which the latter impose an inappropriate responsibility on the individual, which is additionally particularly often accompanied by public stigmatization. On the other hand, they contribute to a self-critical reflection in the context of diagnosing, which knows about the sociostructural embedding of diagnoses and their effects as well as the stigmatizations, discriminations and oppressions possibly associated with them, and may include this knowledge in the elaboration of an appropriate diagnosis and therapy concept.

## Conclusion

Health care does not take place in a “social vacuum” [70]. Mental health disparities exist among various social categories, and intersectionality as a framework may help research, policy-making, and mental health care professionals identify and address these unjustified disparities by examining how multiple social identities intersect to create unique experiences and life opportunities molded by stigmatization, discrimination and oppression. Intersectional approaches may help to address the complexity, interdependence, and mutual constitution of social inequalities better than previous approaches that examine only one axis of sociostructural stratification by helping to identify the needs of those at greatest risk of poor health. Intersectional approaches may be of particular importance in the mental health field, as patients are already more likely to be exposed to stigma and discrimination on the basis of their illness and may thus be particularly effective in terms of socially and structurally sensitized diagnosis and treatment. It should also be borne in mind here that the reduction of a person to typical social identity characteristics is misguided if it is not assessed in terms of its justification and relevance in the individual case. However, by addressing existing health disparities, particularly for multiple disadvantaged individuals, stakeholders can contribute not only to better address the needs of multiple disadvantaged individuals with mental health issues but also to the promotion of equity in the field of mental health.

## Data Availability

Not applicable.

## Abbreviations

ADHD: Attention Deficit Hyperactivity Disorder (ADHD)
MDD: Major Depressive Disorder
PTSD: Post-traumatic Stress Disorder
DSM-III: Diagnostic and Statistical Manual of Mental Disorders III
DSM-IV: Diagnostic and Statistical Manual of Mental Disorders IV

## Footnotes

1 The term “*race*” refers to the social, historically made and changing construct of dividing people into groups, mostly based on the naturalistic reduction to various physical characteristics. Categorisation by *race* is accompanied by multi-layered social attributions of meaning and practices that can be understood as consequences of racialisation and racism (cf. for this in more detail: [2, 3]). The term “ethnicity” here refers to a group of people who identify with each other on the basis of perceived common characteristics.

2 While the existence of such social stratifications is well-documented, it must be caveated that most research comes from nations in North America, Europe, and Australia (exceptions are e.g. [24]). The complex social conditions that inhibit or reinforce inequalities can hardly be translated internationally, so there is a need for further research in this area. Nonetheless, it is reasonable to assume that discriminatory practices such as those described here can occur in other societies as well, even if the groups involved may be different.

3 All category designations used in the following are taken from the empirical studies cited and have not been adapted in favour of uniformity or the like in order to avoid shifts in meaning from the original studies. In the studies cited, these categories are self-reported ones.

4 While the objection raised in practice that the multiplicity of categories only complicates matters and is deemed to be impractical seems to me to be relevant insofar as useful tools should be developed to assist in the consideration of the different categories, nevertheless the mere fact of the structural complexity of discrimination and the excessive demands arising from it may not justify continuing to condone it.

5 Some of the following remarks on the example of the category “mental health issues” may also be assumed in modified form for other social categories mentioned above, since stigmas and their underlying misconceptions also play an influential role there.

6 Particularly helpful in this regard are the comments of James Livingston, who examines stigma toward people with mental health and substance use issues in more detail and begins by dividing stigma into the three forms of self-stigma, social stigma, and structural stigma; see for example [14]. I thank one reviewer for this valuable reference.

7 A first attempt of a systematic aggregation of marginalized social identity characteristics got attention within the so-called “matrix of domination” [62].
